# Reactive Astrocytosis in a Mouse Model of Chronic Polyamine Catabolism Activation

**DOI:** 10.3390/biom11091274

**Published:** 2021-08-25

**Authors:** Chiara Cervetto, Monica Averna, Laura Vergani, Marco Pedrazzi, Sarah Amato, Simone Pelassa, Stefano Giuliani, Francesca Baldini, Guido Maura, Paolo Mariottini, Manuela Marcoli, Manuela Cervelli

**Affiliations:** 1 Department of Pharmacy, Section of Pharmacology and Toxicology, University of Genova, Viale Cembrano 4, 16148 Genoa, Italy; cervetto@difar.unige.it (C.C.); amato@difar.unige.it (S.A.); simonepelassa@gaslini.org (S.P.); maura@difar.unige.it (G.M.); 2 Department of Experimental Medicine, Section of Biochemistry, University of Genova, Viale Benedetto XV 1, 16132 Genoa, Italy; monica.averna@unige.it (M.A.); marco.pedrazzi@unige.it (M.P.); 3 Department of Earth, Environment and Life Sciences (DISTAV), University of Genova, Corso Europa 26, 16132 Genoa, Italy; laura.vergani@unige.it (L.V.); francesca.baldini@unige.it (F.B.); 4 Department of Science, University of Rome “Roma Tre”, Viale Marconi 446, 00146 Rome, Italy; stefano.giuliani@uniroma3.it (S.G.); paolo.mariottini@uniroma3.it (P.M.); 5 Neurodevelopment, Neurogenetics and Molecular Neurobiology Unit, IRCCS Fondazione Santa Lucia, Via del Fosso di Fiorano 64, 00143 Rome, Italy

**Keywords:** astrocyte processes, nerve terminals, excitotoxicity, oxidative stress, polyamines, spermine oxidase (SMOX)

## Abstract

Background: In the brain, polyamines are mainly synthesized in neurons, but preferentially accumulated in astrocytes, and are proposed to be involved in neurodegenerative/neuroinflammatory disorders and neuron injury. A transgenic mouse overexpressing spermine oxidase (SMOX, which specifically oxidizes spermine) in the neocortex neurons (Dach-SMOX mouse) was proved to be a model of increased susceptibility to excitotoxic injury. Methods: To investigate possible alterations in synapse functioning in Dach-SMOX mouse, both cerebrocortical nerve terminals (synaptosomes) and astrocytic processes (gliosomes) were analysed by assessing polyamine levels, ezrin and vimentin content, glutamate AMPA receptor activation, calcium influx, and catalase activity. Results: The main findings are as follows: (i) the presence of functional calcium-permeable AMPA receptors in synaptosomes from both control and Dach-SMOX mice, and in gliosomes from Dach-SMOX mice only; (ii) reduced content of spermine in gliosomes from Dach-SMOX mice; and (iii) down-regulation and up-regulation of catalase activity in synaptosomes and gliosomes, respectively, from Dach-SMOX mice. Conclusions: Chronic activation of SMOX in neurons leads to major changes in the astrocyte processes including reduced spermine levels, increased calcium influx through calcium-permeable AMPA receptors, and stimulation of catalase activity. Astrocytosis and the astrocyte process alterations, depending on chronic activation of polyamine catabolism, result in synapse dysregulation and neuronal suffering.

## 1. Introduction

Polyamines (PAs) are small, organic polycations that include putrescine (Put), spermidine (Spd), and spermine (Spm). The simplest polyamine, Put, is synthesized from ornithine or arginine, and then is converted to Spd and subsequently to Spm [1]. Spermine oxidase (SMOX) catalyses the back-conversion of Spm into Spd with the production of hydrogen peroxide [2].

Although Spd and Spm are synthesized in neurons, they are preferentially accumulated in glial cells.

The more accepted hypothesis [3,4] is that, after synthesis, PAs are released from neurons into the extracellular space, possibly through synaptic neuronal vesicles, and then accumulate in glial cells [5,6]. In the brain, an imbalance of PA synthesis and flux might alter the neuron–glial communication and activate a pro-inflammatory state [4], suggesting the role of endogenous PAs in maintaining intercellular neuron–astrocyte cross-talk and in neuroprotection [6].

The activation of polyamine metabolism in a mouse model overexpressing SMOX in neo-cortex neurons, the Dach-SMOX model [7,8], has been shown to increase vulnerability to excitotoxic/oxidative insult and cortical epileptogenic activity [9,10,11]. This model of chronic activation of PA catabolism has been used to understand the roles of PAs in central nervous system (CNS) disorders involving alterations of neurotransmission and gliotransmission, and to shed light on roles of astrocytes in vulnerability to neuron injury [9]. Notably, our previous findings indicate the following main changes in the cerebral cortex of the mice undergoing chronic activation of PA catabolism: (i) astrocyte activation (reactive astrocytosis) and neuron loss; (ii) increased susceptibility to kainate-evoked cortical epileptogenic activity depending on astrocyte activation; (iii) appearance of a glutamate-releasing response to kainate in astrocyte processes owing to activation of Ca^2+^-permeable α-amino-3-hydroxy-5-methyl-4-isoxazolepropionicacid (AMPA) receptors; (iv) increased oxidative stress and activation of anti-oxidant mechanisms; and (v) alteration in glutamate uptake and efflux through the x_c_^-^ transporter system in astrocytes [9,10,11].

Despite that SMOX overexpression occurs in Dach-SMOX neurons, the main alterations have been observed in glial cells, which might appear puzzling. However, it has to be underlined that neuron and astrocyte actions, at synaptic level, are strictly related, and this has led to move from a neuronocentric to a more comprehensive neuro-astrocentric model for the functioning of CNS. In this regard, the tripartite synapse [12] refers to a bidirectional communication between astrocytes and neurons. Here, glutamatergic nerve terminals release glutamate acting on themselves and on astrocyte processes; in the meantime, perisynaptic astrocyte processes, besides controlling glutamate metabolism and uptake, can also release glutamate, which in turn can affect nerve terminals and trigger signaling cascades [13,14]. In fact, astrocytes sense neural activity at synaptic level and communicate with neurons regulating synaptic function and plasticity [15]. On the other hand, reactive astrocytes may exert both beneficial and detrimental effects on neurons ([16,17] and references therein). In Dach-SMOX mice, both reactive astrocytosis and activation of glutamate release from astrocyte processes seem to contribute, together with increased reactive oxygen species (ROS) production, to the vulnerability to kainate excitotoxicity and epileptogenic activity [9,10].

The main aim of our work was to investigate if and how the altered polyamine catabolism might affect synaptic transmission by focusing on neuronal and astrocytic counterparts of the synapse. In this regard, we investigated the function of isolated cerebrocortical nerve terminals (synaptosomes) and astrocyte processes (gliosomes) prepared from adult Dach-SMOX mice. Isolated synaptosomes indeed preserve the features of in situ nerve terminals, and reflect the behaviour of the presynaptic terminals in neuron–astrocyte networks [18,19,20], while gliosomes preserve the features and reflect the behaviour of the astrocyte processes in the networks [19,21,22]. In synaptosomes and gliosomes from Dach-SMOX and control mice, we assessed the PA content, the oxidative stress markers, and the influx of calcium in response to glutamate receptor activation.

## 2. Materials and Methods

### 2.1. Animals

Male mice of ten months old conditionally overexpressing SMOX in the neo-cortical neurons, with a CD1 background (JoSMOrec mice referred to as Dach-SMOX in the text; [7,10]), and their control syngenic littermates (control in the text) were housed at the Stazione per la Tecnologia Animale at the University of Tor Vergata, Rome, at constant temperature (22 ± 1 °C) and relative humidity (50%) under a regular light–dark schedule (light 07:00–19:00). Food and water were ad libitum. Mice were euthanized by cervical dislocation, followed by decapitation. Animals were processed at the animal care facility of the Dept. of Pharmacy, University of Genova. Animal care and the experimental procedures complied with current laws: the European Communities Parliament and Council Directive of 22 September 2010 (2010/63/EU) and with the Italian DDL 26/2014, in accordance with Decreto Ministeriale 116/1992. Formal approval of these experiments was obtained from the Italian Ministry of Health with the approved protocol No. 964/2015-PR and from the OPBA-University of Genova, No. 30/11/2016. All efforts were made to minimize the number of animals and their suffering, in line with the 3Rs rules (replacement, refinement, and reduction).

### 2.2. Preparation of Purified Nerve Terminals and Astrocyte Processes

The cerebral cortices were quickly removed and placed in ice-cold medium. Gliosomes (purified astrocyte processes) and synaptosomes (purified nerve terminals) were prepared according to the protocol of Nakamura [23], with a few modifications as previously reported [9,19,22]. Briefly, the cerebral cortex was homogenised using a glass-teflon tissue grinder (clearance 0.25 mm) in 10 volumes of sucrose (0.32 M) in Tris buffer (10 mM; pH 7.4). The homogenate was centrifuged (5 min, 1000× *g* at 4 °C) to remove nuclei and debris, and the supernatant was stratified on a discontinuous Percoll’s gradient (2, 6, 10, and 20% *v/v* in Tris-buffered sucrose). After centrifugation (5 min, 33,500× *g* at 4 °C), the gliosomes were collected at the layer between 2% and 6% (*v/v*) Percoll/Tris-buffered sucrose, and synaptosomes at the layer between 10% and 20%. Gliosomes are a purified preparation of astrocyte processes containing vesicles loaded with gliotransmitter, competent for gliotransmitter secretion [9,19]. Synaptosomes are a purified preparation of nerve terminals, negligibly contaminated by astrocytic, microglial or oligodendroglial particles, competent for the release of neurotransmitters in response to different stimulations [20,24,25]. Gliosomes and synaptosomes were washed by centrifugation, and the pellets were resuspended in standard physiological medium buffered with HEPES or in loading buffer for the WB analysis. The HEPES medium had the following composition (mM): NaCl, 128; KCl, 3; MgSO_4_, 1.2; CaCl_2_, 1.2; NaH_2_PO_4_, 1.2; NaHCO_3_, 5; HEPES, 10; glucose, 10; pH 7.4.

### 2.3. Western Blot

Aliquots of total crude preparations (the supernatants collected before stratification on the Percoll gradient, containing both synaptosomes and gliosomes), obtained from both control and SMOX mice, were lysed in Laemmli’s sample buffer by sonication and heated for 5 min at 95 °C. Total proteins (25 μg/lane) were then separated by 8% SDS-PAGE, followed by Western blot. Nitrocellulose membrane was blocked by incubation for 1 h at room temperature with 5% skim milk powder in PBS, containing 0.05% Tween-20. Successively, the membrane was incubated for 16 h at 4 °C with one of the following primary antibodies: anti-vimentin (1:500, Sigma-Aldrich, Milano, Italy), anti-ezrin (1:2000, Sigma-Aldrich), or anti-β-actin (1:1000, Santa Cruz Biotech, Dallas, USA). Suitable peroxidase-conjugated secondary antibodies (1 h at 22 °C) were used. Immunoreactive signals were developed using ECL Select™ Western Blotting Detection Reagent, acquired and quantified using ChemiDoc™ XRS equipped with Quantity One Image Software 4.6.1 (Bio-Rad Laboratories Srl, Segrate, Italy). The membrane was stripped and re-probed.

### 2.4. Polyamine Content

PA concentration was determined in cerebrocortical purified preparations of nerve terminals and astrocyte processes from Dach-SMOX and control mice, as described in [26] with minor modifications. Perchloric acid suspension (5%), supplemented with 1.7-diaminoeptane 100 µM as an internal standard, was added to the cortex samples collected from control and Dach-SMOX mice. Samples were then sonicated in ice with Sonics Vibra-Cells to disintegrate the tissue and centrifuged at 16,100× *g* for 10 min. The supernatant was mixed with saturated Na_2_CO_3_ and then with acetone Dansyl chloride solution (7.5 mg/mL). The mixture was incubated overnight, protected from light at room temperature. The next day, the samples were centrifuged at 16,100× *g* for 15 min at 4 °C; proline solution (5%) was added to the supernatant to remove the unreacted Dansyl chloride. After 30 min, PAs were extracted with toluene (100%) with vigorous vortexing and then rested for 5 min at room temperature in the dark. The organic phase was dried in a 3 Speedvac Concentrator (Savant Instrument, Inc., New York, NY, USA). The dried Dansyl derivatives were stored at −20 °C or dissolved in methanol and immediately assayed. High-performance liquid chromatography technique using the Agilent 1050 system (Agilent Technologies, Santa Clara, CA, USA) was used to detect the PA content, with an Agilent 1050 photodiode type detector. Continuous on-line quantification of chromatographic peaks was carried out by the fluorimeter Agilent 1200 Spectra-Physics Model SP 4290 and the computing program software “Agilent ChemStation”. The separation of Dansyl derivatives was performed on C18 Hypersil BDS 250 mm × 4.6 mm at constant room temperature of 22 °C ± 1. Two mobile phases were used: (A) water/acetonitrile/methanol (50%:30%:20%) and (B) acetonitrile/methanol (60%:40%), with the following elution program: 0–5 min: 72% A–28% B; 5-47 min: 72% A–28% B; 47–50 min: 36%A–64% B; 50–55 min: 20% A–80% B; 55–56 min: 15% A–85% B; 56–75 min: 72% A–28% B at flow rate of 1 mL/min. PA content was normalized using protein concentration measured with the Bradford method [27]; the amount of endogenous PA content was expressed as pmol/µg protein.

### 2.5. Catalase Activity

Enzyme-specific activity of catalase was determined in cerebrocortical purified nerve terminals and astrocyte processes prepared from Dach-SMOX and control mice. Catalase (CAT) activity was evaluated following the consumption of hydrogen peroxide (H_2_O_2_) at 240 nm at 25 °C [28]. To initiate the reaction, aliquots of gliosomes or synaptosomes were suitably diluted in 50 mM PBS (pH 7.8) followed by the addition of H_2_O_2_ (30 mM final concentration). Catalase-specific activity was expressed as millimoles of decomposed H_2_O_2_ per minute per milligram of sample protein. Protein content was determined using the bicinchoninic acid (BCA) assay using bovine serum albumin as a standard [29].

### 2.6. [Ca ^2+^]_i_ Assay

[Ca^2+^]_i_ was determined as previously described [30]. Briefly, purified nerve terminals and astrocyte processes were washed once in HEPES buffer and then incubated in the same buffer containing 10 μM Calcium Green™-1 AM (CG). After 30 min at 37 °C, synaptosomes and gliosomes were washed twice with HEPES buffer, transferred to a black 96-well microplate (50 µg/well), and then exposed to the AMPA receptor agonist (*S*)-CPW399 (100 µM). The fluorescence intensity (excitation 485 nm and emission 535 nm) was measured every 10 s for 10 min using the top reading mode in the fluorescence multilabel reader LB 940 Mithras (Berthold Technologies, Baden Württemberg, Germany). Variations of the fluorescence values were calculated as the difference between each fluorescence value recorded and that measured at time zero. The values obtained were then subtracted from the relevant control values. Alternatively, nerve terminals and astrocyte processes were exposed to the AMPA receptor agonist (*S*)-CPW399 (100 µM) in the absence or in the presence of the selective antagonist of the GluA2-lacking Ca^2+^-permeable AMPA receptors, 1-naphthylacetyl spermine trihydrochloride (NASPM) (50 µM). The fluorescence intensity was measured every 10 s for 4 min. For the determination of Ca^2+^ flux, the area underlying each curve was measured by means of the public image processing program ImageJ (NIH and LOCI University of Wisconsin).

### 2.7. Statistical Analysis

Each assay was performed in triplicate. Unless otherwise indicated, data are expressed as mean ± standard deviation (S.D). The significance of the difference was analyzed by the Mann–Whitney test; a probability of *p* ≤ 0.05 was taken as the limit for statistical significance.

The statistical significance of the differences in polyamine content between Dach-SMOX and control animals was determined using the one-way ANOVA test (GraphPad Prism, San Diego, CA, USA) with Tukey post hoc test. A *p*-value of <0.05 was accepted as indicative of a statistically significant difference.

### 2.8. Chemicals

All chemicals, unless otherwise indicated, were of analytical grade and were obtained from Sigma-Aldrich Corp. (Milano, Italy). (*S*)-CPW399, and 1-naphthylacetyl spermine trihydrochloride were from Tocris Cookson (Bristol, UK). Drugs were dissolved in distilled water or in physiological medium. Calcium Green™-1AM was purchased from Life Technologies Italia (Milano, Italy).

## 3. Results

### 3.1. Analysis of Ezrin and Vimentin Levels in the Cerebral Cortex of Dach-SMOX and Control Mice

**Figure 1:**
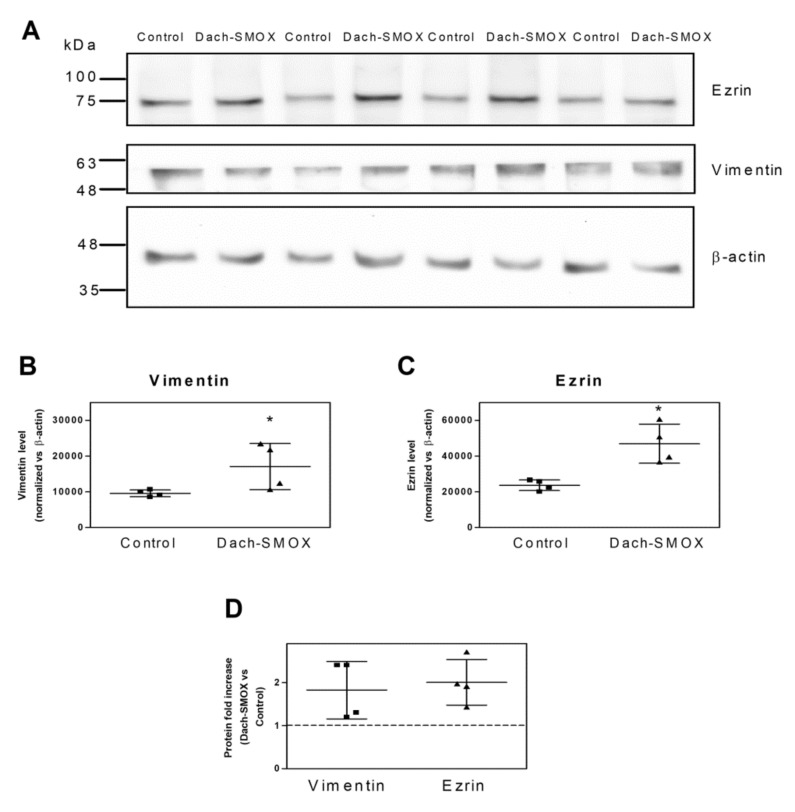
Increased levels of ezrin and vimentin in Dach-SMOX cerebral cortex. Western blot analysis on cerebral cortex homogenate from Dach-SMOX and control mice using antibodies against ezrin and vimentin. (**A**) Aliquots (25 µg/lane) of total crude preparations, containing both synaptosomes and gliosomes, were submitted to 8% SDS-PAGE followed by Western blot for ezrin and vimentin. The membrane was stripped and re-probed for β-actin. Four controls, as well as four Dach-SMOX mice, were analyzed. Protein standard molecular weights are reported (kDa). (**B**,**C**) The relevant immunoreactive bands were quantified and normalized versus β-actin. Data are reported as filled squares (control) and filled triangles (Dach-SMOX). Mean and SD are also reported (*n* = 4). * *p* ≤ 0.05, according to Mann-Whitney test. (**D**) The fold increase for ezrin and vimentin in Dach-SMOX mice is shown. Data are reported as filled squares (vimentin) and filled triangles (ezrin). Mean and SD are also reported (*n* = 4).

As it has been reported that cytoskeletal protein expression can be modulated in neuroinflammation and reactive astrocytosis [16,17,31,32], and astrogliosis has been described as a feature of Dach-SMOX mice [7,9], we analyzed ezrin and vimentin protein levels in both Dach-SMOX and control mice. WB was performed on the supernatant obtained after homogenization of the cerebral cortex, which contained both the nerve terminals and the astrocyte processes (see the WB bands in Figure 1A). We found levels of both vimentin (Figure 1B) and ezrin (Figure 1C) in Dach-SMOX to be significantly higher than those detected in control mice. Specifically, as shown in Figure 1D, we observed an approximately twofold increase of the two protein levels (2.0 ± 0.5 for ezrin, 1.8 ± 0.7 for vimentin) in Dach-SMOX with respect to control mice.

These findings indicate that, in the Dach-SMOX cerebral cortex, there is an increase in protein levels associated with a neuroinflammatory condition, confirming the presence of reactive astrogliosis in Dach-SMOX mice.

### 3.2. Polyamine Content in Purified Nerve Terminals and Astrocyte Processes

**Figure 2:**
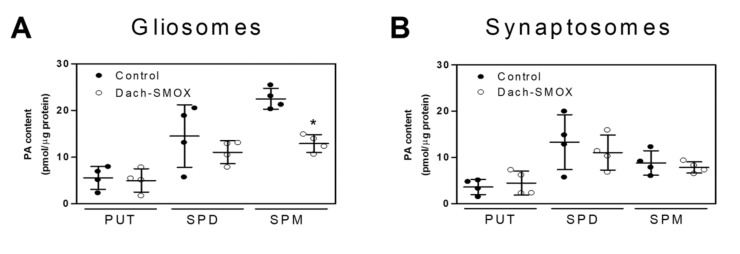
Significant reduction of Spm content in Dach-SMOX cerebrocortical gliosomes. Put, Spd, and Spm content in gliosomes and synaptosomes from Dach-SMOX and control mice. For details, see Materials and Methods. Filled circles represent the PA content (pmol/µg protein) in control (black) and Dach-SMOX (white) gliosomes (**A**) and synaptosomes (**B**). Mean and SD are reported (*n* = 4). * *p* < 0.05 according to one-way ANOVA test and post hoc test Tuckey.

Dach-SMOX mice showed a lower content of Spm in gliosomes compared with the control mice; on the contrary, no differences were observed in the Spm content in synaptosomes (Figure 2). Meanwhile, no changes occurred in the Spd and Put levels in gliosomes and synaptosomes of both Dach-SMOX and control mice. Notably, in control mice, the Spm level was higher in gliosomes than in synaptosomes (22.5 ± 2.3 pmol/μg protein in gliosomes and 8.8 ± 2.6 pmol/μg protein in synaptosomes, mean ± SD of four experiments; *p* < 0.01).

These findings in mice overexpressing SMOX, which specifically oxidizes Spm and plays a dominant role in brain PA catabolism, indicate that the PA metabolism at synaptic level undergoes an important homeostatic control. Unexpectedly, in Dach-SMOX mice, the only change in PA content at synapse level was a reduction in Spm in gliosomes.

### 3.3. [Ca^2+^]_i_ Evaluation in Purified Nerve Terminals and Astrocyte Processes

**Figure 3:**
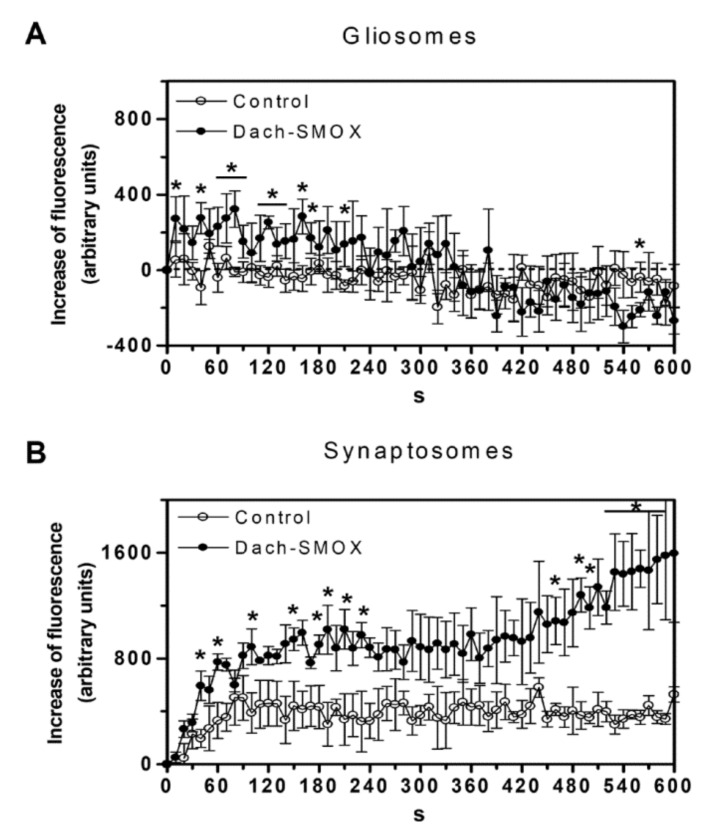
AMPA receptor activation-evoked [Ca^2+^]_i_ increase in Dach-SMOX cerebrocortical gliosomes and synaptosomes. CG-loaded particles (gliosomes or synaptosomes) from control (○) and Dach-SMOX (●) mice were treated with 100 µM CPW399 for the indicated time at 37 °C. CG-dependent fluorescence was monitored every 10 s from 0 to 600 s. [Ca^2+^]_i_ increase is expressed as “increase of fluorescence”, which is the difference between the CG-dependent fluorescence of the stimulated samples and the ones of the vehicle-treated samples, both measured at each recording time and subtracted by the one measured at the starting time. (**A**) Data are means ± SEM from three (●) or four (○) independent experiments in triplicate. (**B**) Data are means ± SEM from three (●,○) independent experiments in triplicate. * *p* ≤ 0.05, according to Mann–Whitney test.

As it has been previously reported [9] that chronic activation of PA catabolism involves the engagement of Ca^2+^-permeable AMPA receptors in the cerebral cortex, we analyzed [Ca^2+^]_i_ changes following stimulation of this receptor triggered by (S)-CPW399 in either gliosomes and synaptosomes purified from both Dach-SMOX and control mice. As shown in Figure 3A, a [Ca^2+^]_i_ increase evoked by the AMPA receptor agonist (S)-CPW399 was detectable in gliosomes only from Dach-SMOX mice. Moreover, after 300 s, the responding gliosomes were able to restore [Ca^2+^]_i_ to basal level, suggesting their ability to buffer [Ca^2+^]_i_ increase following AMPA receptor stimulation. Furthermore, the ostensible decrease after 300 s was not statistically significant (except only for the point at 560 s). On the other hand, synaptosomes from both control and Dach-SMOX mice were sensitive to (*S*)-CPW399 (Figure 3B), although the extent of the [Ca^2+^]_i_ increase after 240 s was 1.7-fold higher in Dach-SMOX than in control synaptosomes, a ratio that increases to 2.6-fold after 600 s, indicating disruption of intracellular calcium homeostasis. Furthermore, we cannot exclude that the bi-phasic increase in Ca^2+^ level in synaptosomes from Dach-SMOX mice might reflect an increased release of signalling molecules (e.g., glutamate), which in turn can induce a further increase in [Ca^2+^]_ì_.

**Figure 4:**
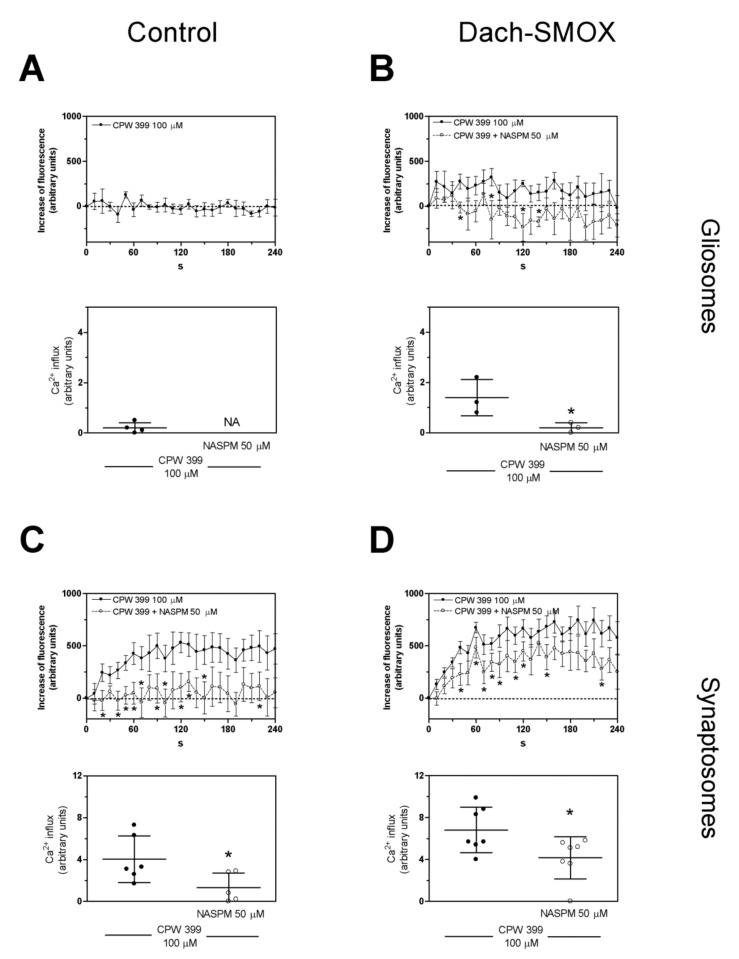
Antagonism by NASPM of the [Ca^2+^]_i_ influx evoked by AMPA receptor activation in Dach-SMOX cerebrocortical gliosomes and synaptosomes. CG-loaded particles (gliosomes or synaptosomes) were treated with 100 µM CPW399 in the absence (●) or in the presence (○) of the selective antagonist of GluA2-lacking AMPA receptor NASPM (50 µM) for the indicated time at 37 °C. CG-dependent fluorescence was monitored every 10 s from 0 to 240 s. [Ca^2+^]_i_ increase is expressed as “increase of fluorescence”. The areas reported in each upper panel were quantified to estimate the calcium influxes. (**A**) [Ca^2+^]_i_ increase (upper panel) and calcium influx (lower panel) in gliosomes from control mice. Data are means ± SEM from four (●) independent experiments in triplicate. NA, not assessed. (**B**) [Ca^2+^]_i_ increase (upper panel) and calcium influx (lower panel) in gliosomes from Dach-SMOX mice. Data are means ± SEM from three (●,○) independent experiments in triplicate. (**C**) [Ca^2+^]_i_ increase (upper panel) and calcium influx (lower panel) in synaptosomes from control mice. Data are means ± SEM from six (●) or five (○) independent experiments in triplicate. (**D**) [Ca^2+^]_i_ increase (upper panel) and calcium influx (lower panel) in synaptosomes from Dach-SMOX mice. Data are means ± SEM from seven (●, ○) independent experiments in triplicate. * *p* ≤ 0.05, according to Mann–Whitney test.

We evaluated the effect of NASPM, a selective antagonist of Ca^2+^-permeable GluA2-lacking AMPA receptors, on the calcium influx detected within 240 s. As shown in Figure 4A,B, the increase of [Ca^2+^]_i_ in gliosomes, detectable only in Dach-SMOX, was abrogated in the presence of NASPM, suggesting that Ca^2+^-permeable AMPA receptors accounted for the observed [Ca^2+^]_i_ increase. Moreover, the (*S*)-CPW399-evoked [Ca^2+^]_i_ increase in synaptosomes (Figure 4C,D) was significantly inhibited, but not fully prevented, by NASPM, specifically by 67% in control and by 39% in Dach-SMOX, possibly indicating the contribution of different receptors or channels.

Altogether, these findings indicate the presence of functional Ca^2+^-permeable AMPA receptors in the astrocyte processes from Dach-SMOX, but not from control mice; conversely, the receptors appeared to function in the nerve terminals from both control and Dach-SMOX mice. Moreover, in Dach-SMOX synaptosomes, the finding suggests a dysregulation of [Ca^2+^]_i_ following the receptor activation, compatible with neuron suffering.

### 3.4. Oxidative Stress in Nerve Terminals and Astrocyte Processes from Dach-SMOX Cortex

**Figure 5:**
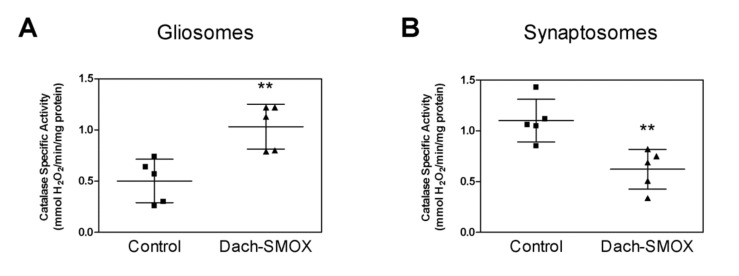
Analysis of catalase activity in Dach-SMOX cerebrocortical gliosomes and synaptosomes. The specific activity of catalase (mmoles of decomposed H_2_O_2_ per min per mg protein) was quantified by spectrophotometric assay in brain cortex (**A**) gliosomes and (**B**) synaptosome from both control and Dach-SMOX mice. Data are reported as filled squares (control) and filled triangles (Dach-SMOX). Mean and SD of experiments performed in quadruplicate on five animals per group are also reported. ** *p* ≤ 0.01, according to Mann–Whitney test.

As a marker of oxidative stress, the specific activity of the antioxidant enzyme catalase was evaluated in both synaptosomes and gliosomes purified from the cortex of both Dach-SMOX and control mice (Figure 5). In the astrocytes processes from Dach-SMOX mice, we observed stimulation of the catalase activity that was almost doubled with respect to control mice. Figure 5A shows, for the catalase, a mean specific activity of about 0.5 ± 0.23 mmoles H_2_O_2_ consumed/min/mg protein in gliosomes from control mice, while it significantly increased to 1.03 ± 0.22 mmoles H_2_O_2_ consumed/min/mg protein in the Dach-SMOX gliosomes, with an increase of about 106% (*p* ≤ 0.01).

An opposite result was observed for catalase activity in the nerve terminals, where the catalase activity was almost halved in synaptosomes from Dach-SMOX mice with respect to the control. Figure 5B shows, for the catalase, a mean specific activity of about 1.09 ± 0.22 mmoles H_2_O_2_ consumed/min/mg protein in synaptosomes from control mice, which was significantly reduced to 0.63 ± 0.21 mmoles H_2_O_2_ consumed/min/mg protein in the Dach-SMOX synaptosomes, with a decrease of about 42% (*p* ≤ 0.01).

The findings indicating the stimulation of catalase activity in gliosomes from Dach-SMOX mice suggest the activation of the defense mechanisms against oxidative stress, compatible with a reactive astrocytosis condition. On the other hand, the down-regulation of catalase activity in synaptosomes from Dach-SMOX mice might indicate an impaired defense against oxidative stress, compatible with damage of the nerve terminals.

## 4. Discussion

The main novel findings are summarized as follows.

### 4.1. Increased Levels of the Astroglial Markers Ezrin and Vimentin

The increased levels of the astroglial markers ezrin and vimentin were measured for the first time in the cerebral cortex of Dach-SMOX mice and indicate reactive astrocytes and a neuroinflammation condition [31]. Indeed, vimentin is a marker for reactive astrocytes [16,17], as it may regulate both the beneficial and detrimental effects of reactive astrocytes at synapses [32]. Ezrin, preferentially localized in the fine perisynaptic astrocytic processes (PAPs), is required for their motility and the regulation of synapse coverage [33,34], and it is mechanistically involved in the structural changes related to astrocyte activation linked to a response to injury [33]. In fact, the increase in ezrin cannot be solely considered a sign of reactive astrocytosis, but, considering its presence in PAPs, it specifically indicates changes in these fine processes, with relevant consequences on glia–synaptic interactions. Altogether, the findings reported in the previous and the present paper indicate a remodeling of astrocytes and of perisynaptic astrocyte processes in response to chronic activation of PA catabolism, with an increased ramification of the astrocytes and enrichment in the astroglial markers GFAP, ezrin, and vimentin, which are typical of reactive astrocytes.

### 4.2. Altered Calcium Responses in Astrocyte Processes and Nerve Terminals

Gliosomes from Dach-SMOX mice expressed functional Ca^2+^-permeable AMPA receptors; the finding perfectly matches the Ca^2+^-dependent release of glutamate from the astrocyte processes upon activation of GluA2-lacking AMPA receptors, a response that was completely lacking in control astrocyte processes [9]. The presence of functional AMPA receptors in the processes appears to be well consistent with the decrease in Spm content in the processes, as intracellular Spm is well known to inhibit the Ca^2+^-permeable AMPA receptor functioning. Ca^2+^ entry through these AMPA receptors appears to be relevant to the topic of Ca^2+^ microdomains, of the dynamic Ca^2+^ changes spatially restricted to fine perisynaptic astrocytic processes [35,36], and of their changes in reactive astrocytosis.

Moreover, synaptosomes from both Dach-SMOX and control mice expressed functional Ca^2+^-permeable AMPA receptors; however, the [Ca^2+^]_i_ increase seems to be dramatically dysregulated in nerve terminals of Dach-SMOX mice, perhaps not only as a result of an emphasized biophase. Dysregulation of calcium homeostasis in nerve terminals, together with the down-regulation of catalase activity, could indicate, and contribute to, neuron suffering in Dach-SMOX mice. Consistently, a reduction in the number of neurons and in the relative abundance of nerve terminals has been reported in the cerebral cortex of the Dach-SMOX mice [9].

### 4.3. Reduction of Spermine in Astrocyte Processes, but Not in Nerve Terminals

Dach-SMOX mice display a lower content of Spm only in gliosomes, but no differences in synaptosomes, compared with control mice. The important role of polyamines in brain plasticity and neurodegenerative processes is well known, and there are several cases of human conditions caused by altered polyamine concentration. A striking example is the low Spm concentration due to a mutation in the X-chromosome responsible for Snyder–Robinson syndrome [37]. Other general disturbance of human brain function occurs when polyamines’ concentration is altered, such as mental retardation, hypotonia and cerebellar dysfunction [38], depression with suicidal tendencies [39], and amyotrophic lateral sclerosis [40]. Furthermore, the elimination of Spm and Spd from the diet leads to the reduction of polyamines’ content, which produces the effect of weakening neuronal resistance to cytotoxic effects [41,42]. These data point out that the decrease in both Spm and Spd levels is clearly involved in the pathology of neurodegenerative processes, as well as in age-related illnesses like Parkinson’s [43] and Huntington’s diseases [44].

Polyamines such as Spm and Spd are involved in glial–neuronal communication, especially during periods of stress such as ischemia and trauma. One of the principal differences between glia and neurons lies in the fact that polyamines are not synthesized, but stored almost exclusively in glial cells, from which they can be released to regulate neuronal synaptic activity [5,6].

Interaction of Spm and Spd with various receptor- and voltage-gated cationic channels may regulate cation flux in excitable tissues and control the synaptic plasticity, in which involvement of the NMDA receptor has been amply demonstrated in the past [3,45,46]. Moreover, the postsynaptic responses of both NMDA [47] and non-NMDA (AMPA and kainate) receptors can be regulated by Spm and Spd release [3]. Extracellular Spm can modulate synaptic transmission in vivo, increasing the extent of NMDA receptor currents potentiating the postsynaptic sensitivity to excitatory inputs [3,48,49,50,51]. Of note, intracellular Spm can cause rectification of the AMPA and kainate receptors by blocking the pore of the receptor channel to prevent the flux of Na^+^ and Ca^2+^ as the membrane is depolarized. Only a subset of AMPA and kainate receptors, expressed on particular types of neurons and glial cells, display inward rectification by intracellular PAs. In particular, Spm causes rectification of Ca^2+^-permeable receptors that either lack GluA2 and GluA6 or contain the unedited (Q) forms of these subunits [50]. Both Spm and Spd are released from neurons or glia and rapidly re-incorporated into those cells; in particular, Spd is more efficiently transported into glial cells than Spm, but Spm is more efficiently transported into synaptic vesicles and synaptosomes [4].

The lower content of Spm in gliosomes of Dach-SMOX mice is in line with previous studies indicating that glial cells are responsive to the chronicle oxidative stress from which the Dach-SMOX mice are suffering [9,10]. It has been observed that the excitability threshold at synapses containing polyamine-sensitive AMPA receptors is affected by changes in intracellular PA levels that alter Ca^2+^ flux [50]. The lower content of Spm in glial cells can be responsible for rectification of Ca^2+^-permeable GluA2-lacking receptors by a reduced block, and of a receptor-evoked Ca^2+^ influx, highlighting the presence of this subset of AMPA receptors only in the astrocyte processes of this mouse line.

The low level of Spm in glia cells can be explained by hypothesizing that astrocytes excrete Spm via connexin gap junctions or/and organic cation transporter [5,6] to replenish neurons depleted of Spm and keep constant the concentration of this molecule as well as the right Spm/Spd balance within neuronal cells.

### 4.4. Stimulation of Antioxidant Defense in Astrocyte Processes, and Impairment in Nerve Terminals

As SMOX catalyses the oxidation of Spm into Spd with the production of hydrogen peroxide, Dach-SMOX mice present an increased vulnerability to excitotoxicity owing to an altered Spm/Spd ratio and an augmented oxidative stress [7].

An imbalance in the brain redox state, due to increased production of ROS or to failure of the antioxidant systems, has been described in many neurodegenerative diseases [52]. The consequent oxidative stress condition may result in selective neuronal cell damage and dysfunction. Therefore, from SMOX over-activity, an increased production of hydrogen peroxide and ROS-mediated damage was reported [7]. Indeed, oxidative stress is a physio-pathological condition involved in many neurodegenerative diseases such as Alzheimer’s and Parkinson’s diseases [53,54]. In the brain, an excess of ROS may trigger reactions of lipid peroxidation, but the presence of antioxidant enzymes such as catalase protect neurons and glial cells. According to expectations, in astrocyte processes of Dach-SMOX mice, we observed a stimulation of catalase activity to degrade the excess hydrogen peroxide produced by SMOX over-activity, and this result well fits with the reduced Spm level. Conversely, in synaptosomes of Dach-SMOX animals, we observed a reduction in catalase activity and no difference in Spm level.

This is consistent with a condition of astrogliosis, which may be triggered by persistent oxidative stress. On the other hand, the reduced activity of catalase in nerve terminals from Dach-SMOX mice might be a sign of neuron damage. In fact, catalase deficiency or malfunctioning was found to be associated with neurodegenerative disorders such as Alzheimer’s and Parkinson’s disease and neuron damage [55]. The functional changes in synaptosomes are in agreement with their loss in the cerebrocortical region that we previously reported in Dach-SMOX mice [9].

### 4.5. Insights on Polyamines and Neuron/Astrocytes Crosstalk in Dach-SMOX Mice

The findings reported here utilizing the *Dach-SMOX* mouse model emphasize the crucial role of PAs in the control of synapse transmission and intercellular communication in healthy brain. We do believe that both change of PA metabolism and concomitant hydrogen peroxide overproduction co-occur in neurons and affect astrocytes.

Astrocytes may become reactive possibly responding to overproduction of hydrogen peroxide owing to SMOX overexpression and/or as a consequence of neuron impairment (altered neuronal activity and possible lack of trophic signalling from neurons) [15]. In turn, reactive astrocytes may determine the detrimental effects on neurons. It is worth noting that the reactive astrocyte processes at the cerebrocortical synapses seem to have a reduced supply of Spm, possibly dampening roles of astrocytes in neuronal protection [6]. Notably, a higher Spm content in control mice gliosomes with respect to synaptosomes is consistent with the reported PA accumulation in glial cells [5,6], also supporting a protective role of glial PAs in healthy neuron–astrocyte networks [6].

Reactive astrocytes might also directly contribute to increase the extracellular glutamate synaptic levels (Table 1).

As summarized in Table 1, at least three mechanisms may be involved in astrocyte-dependent increase of extracellular glutamate and contribution to excitotoxicity: the functioning of glutamate-releasing AMPA receptors ([9] and present results), the functioning of the x_c_^-^ transporter [10], and a likely impaired glutamate clearance owing to reduced expression of the astrocytic excitatory amino acid transporters EAAT1 and EAAT2 [10]. In particular, the reactive astrocyte processes, expressing AMPA receptors, which allow calcium entry and activate glutamate release when responding to glutamate itself, may participate to a positive feedback loop. All these mechanisms may result in a chronic condition of excitotoxicity that, together with oxidative stress, might result in neuron vulnerability. As a matter of fact, neurons, as well as the nerve terminals, are reduced in numbers in SMOX mice, and the nerve terminals appear to be suffering, with defective ability to control the intracellular calcium responses to glutamate receptor activation, and depletion of catalase; both of these conditions can be linked to a reduced capability to cope with excitotoxic and oxidative insults. Indeed, when the AMPA receptors were activated by kainate in Dach-SMOX cerebrocortical slices, an epileptogenic activity was found, which was to be dramatically reduced by reducing the astrocyte contribution [9]. Consistently, blocking the x_c_^-^ transporter obliterated the increased susceptibility of SMOX mice to epileptogenic activity [10]. Therefore, Dach-SMOX mice can be proposed as a suitable chronic model that would help to understand vulnerability of neurons to oxidative stress/excitotoxicity. Moreover, our findings emphasize the role that the PA system has in glial cells and strongly suggests the possibility that PAs can be mediators of a cross-talk between neurons and astrocytes, required to maintain the physiological integrity of the glutamatergic synapse and to protect neurons from oxidative and excitotoxic damage.

## 5. Conclusions

In conclusion, the findings using the Dach-SMOX genetic model specifically show for the first time the central role of Spm in the neuron/astrocyte cross-talk. Astrocytes acting as polyamine accumulating cells respond to SMOX overexpression in neurons in an attempt to regulate polyamine concentration resulting in reactive astrocytosis. In turn, dysfunctional astrocytes activate calcium exchange from fine perisynaptic processes via GluA2-lacking AMPA receptor channels. As synthesis and degradation pathways of PAs are involved in many CNS diseases and play a major role in life span, these new insights could help in understanding the role of PAs in brain and their metabolites on glial and neuronal function.

## Figures and Tables

**Table 1:** Astrocyte-dependent mechanisms potentially contributing to excitotoxicity in Dach-SMOX mice.

Mechanism	Type of Change	Consequence	Ref
Ca^2+^-permeable AMPA receptor	expression of functional	glutamate release	[9]
X_c_^-^ transporter	increased function	glutamate release (in-outside transport)	[10]
EAAT1, EAAT2	reduced expression	impaired glutamate clearance	[10]

## Data Availability

Data available on request from the corresponding authors.
